# Whole-Genome Shotgun Metagenomic Sequencing Reveals Distinct Gut Microbiome Signatures of Obese Cats

**DOI:** 10.1128/spectrum.00837-22

**Published:** 2022-04-25

**Authors:** Xiaolei Ma, Emily Brinker, Emily C. Graff, Wenqi Cao, Amanda L. Gross, Aime K. Johnson, Chao Zhang, Douglas R. Martin, Xu Wang

**Affiliations:** a Department of Pathobiology, College of Veterinary Medicine, Auburn University, Auburn, Alabama, USA; b School of Life Sciences and Technology, Tongji University, Shanghai, China; c Scott-Ritchey Research Center, College of Veterinary Medicine, Auburn University, Auburn, Alabama, USA; d Department of Clinical Sciences, College of Veterinary Medicine, Auburn University, Auburn, Alabama, USA; e Department of Plastic and Reconstructive Surgery, Shanghai Ninth People’s Hospital, Shanghai Institute of Precision Medicine, Shanghai Jiao Tong University School of Medicine, Shanghai, China; f Department of Anatomy, Physiology and Pharmacology, College of Veterinary Medicine, Auburn University, Auburn, Alabama, USA; g Center for Advanced Science, Innovation and Commerce, Alabama Agricultural Experiment Station, Auburn, Alabama, USA; h HudsonAlpha Institute for Biotechnology, Huntsville, Alabama, USA; South China Agricultural University

**Keywords:** feline obesity, gut microbiota, Firmicutes-to-Bacteroidetes ratio, *Erysipelotrichaceae*, *Bifidobacterium*, *Dialister*, *Olsenella*, *Campylobacter*

## Abstract

Overweight and obesity are growing health problems in domestic cats, increasing the risks of insulin resistance, lipid dyscrasias, neoplasia, cardiovascular disease, and decreasing longevity. The signature of obesity in the feline gut microbiota has not been studied at the whole-genome metagenomic level. We performed whole-genome shotgun metagenomic sequencing in the fecal samples of eight overweight/obese and eight normal cats housed in the same research environment. We obtained 271 Gbp of sequences and generated a 961-Mbp *de novo* reference contig assembly, with 1.14 million annotated microbial genes. In the obese cat microbiome, we discovered a significant reduction in microbial diversity (*P < *0.01) and Firmicutes abundance (*P = *0.005), as well as decreased Firmicutes/Bacteroidetes ratios (*P = *0.02), which is the inverse of obese human/mouse microbiota. Linear discriminant analysis and quantitative PCR (qPCR) validation revealed significant increases of *Bifidobacterium* sp.*, Olsenella provencensis, Dialister sp.CAG:486*, and Campylobacter upsaliensis as the hallmark of obese microbiota among 400 enriched species, whereas 1,525 bacterial species have decreased abundance in the obese microbiome. Phascolarctobacterium succinatutens and an uncharacterized *Erysipelotrichaceae bacterium* are highly abundant (>0.05%) in the normal gut with over 400-fold depletion in the obese microbiome. Fatty acid synthesis-related pathways are significantly overrepresented in the obese compared with the normal cat microbiome. In conclusion, we discovered dramatically decreased microbial diversity in obese cat gut microbiota, suggesting potential dysbiosis. A panel of seven significantly altered, highly abundant species can serve as a microbiome indicator of obesity. Our findings in the obese cat microbiome composition, abundance, and functional capacities provide new insights into feline obesity.

**IMPORTANCE** Obesity affects around 45% of domestic cats, and licensed drugs for treating feline obesity are lacking. Physical exercise and calorie restrictions are commonly used for weight loss but with limited efficacy. Through comprehensive analyses of normal and obese cat gut bacteria flora, we identified dramatic shifts in the obese gut microbiome, including four bacterial species significantly enriched and two species depleted in the obese cats. The key bacterial community and functional capacity alterations discovered from this study will inform new weight management strategies for obese cats, such as evaluations of specific diet formulas that alter the microbiome composition, and the development of prebiotics and probiotics that promote the increase of beneficial species and the depletion of obesity-associated species. Interestingly, these bacteria identified in our study were also reported to affect the weight loss success in human patients, suggesting translational potential in human obesity.

## INTRODUCTION

A combination of excessive food intake and lack of physical exercise leads to an expansion of adipose tissue in the body, resulting in metabolic dysregulation. When excess body adipose tissue has accumulated to the extent that it has adverse effects on health, it is termed obesity. Feline obesity is a major epidemic with a current prevalence of around 45% (1–3) and is considered the second most common health problem in domestic cats in developed countries (4). It is linked to many systemic health conditions, including altered lipid profiles (5), insulin resistance (6), neoplasia, urinary diseases (3), cardiovascular diseases (7), and reduced life span. There are no available licensed drugs for treating feline obesity, and classic interventions for weight loss such as calorie restrictions and physical exercise are often challenging and are ultimately ineffective (8). Understanding the obese cat gut microbiota is necessary to facilitate the development of treatment strategies through dietary probiotics and gut microbiota manipulations.

The gut microbiome is the entire collection of microorganisms in the gastrointestinal tract. In humans, microorganisms are about 38 trillion in total, exceeding the number of human cells (9). The gut microbiota is an integral part of the body, affecting many aspects of disease physiology, including rheumatoid arthritis (10), colorectal cancer (11, 12), cardiovascular disease (13), and inflammatory bowel disease (14, 15). Gut microbiome composition and function are directly related to digestion, nutrient metabolism, and assimilation, which play important modulative roles in total body adiposity. The gut microbiota modulates obesity through food absorption and low-grade inflammation (16, 17). In mice, changes in intestinal bacterial compositions and microbial metabolites can cause increases in endotoxemia and further exacerbate obesity and insulin resistance (18, 19). Studies in both humans and mice have shown that influencing the gut microbiota, such as with fecal transplantation, or external chemicals or drugs, can have favorable or unfavorable effects on fat gain (20–26). Conversely, being overweight or obese can cause dysbiosis, often associated with low microbial diversity and richness in gut microbiota (27–29). Many studies reported that the relative proportions of microbes in the gut microbiota correspond to body weight in humans (30). Obesity can alter the microbial composition in the gut, and reduced levels of Bacteroidetes have been reported in obese versus lean members of twin pairs (29). The reduction of Bacteroidetes in obese animals could be reversed through a calorie-restricted diet (31).

To date, there are over 20 studies on the feline gut microbiota (32–54), all of which used the 16S rDNA sequencing approach. Factors such as diets, pre-/probiotics, age, diarrhea, and other diseased states have been shown to influence gut microbiota composition (34, 35, 37, 55). One study examined the effect of obesity on the gut microbiome and found that the gut microbiome of lean cats was significantly different (*P < *0.05) from that of overweight and obese cats (32). Lean and obese cat gut microbiota were also reported to respond differentially to dietary protein and carbohydrate ratio (48). These previous studies identified phylum and genus level changes in the obese cat microbiome, but failed to discover bacterial species-level changes in the feline gut microbiota. Another limitation is that these studies were often performed using client-owned cats from diverse household environments, which diminished the statistical power to detect microbiome differences. Last but not least, fecal sample collection methods also affect the results of microbiome analysis. Many previous studies collected feces from litterboxes, which could be contaminated, and the microbiota composition can shift after the fecal sample left the intestine. To address these issues and obtain comprehensive genome coverage for bacteria composition at the species level (56), whole-genome shotgun (WGS) metagenomic sequencing was performed in normal versus obese cats, using fecal samples collected from the rectum and descending colon by a fecal loop. We assembled the first cat reference microbial contigs, predicted and annotated taxonomy identity and microbial genes, and discovered and validated significant changes in species abundance in obese versus normal cat gut microbiota. Our results provide a deeper understanding of the feline gut microbiota and its link to body conditions, which shed light on the microbiome basis of feline obesity and will inform the development of weight loss therapy using probiotics and fecal transplantation.

## RESULTS

### A comprehensive characterization of feline gut microbiota using deep WGS metagenomic data.

**FIG 1:**
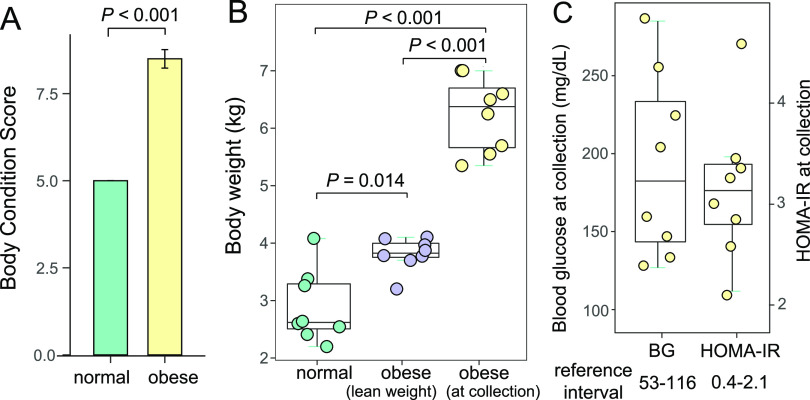
Body weight, body condition score, serum glucose level, and insulin resistance parameters for obese cats. (A) Bar plot of the body condition scores of normal and obese cats in this study. (B) Box plot of the body weight of normal cats, obese cats when they were lean, and obese cats at the time of fecal and blood sample collection. (C) Box plot of the blood glucose scores (left) and homeostatic model assessment for insulin resistance (HOMA-IR) scores (right) of obese cats at collection.

The body condition score (BCS) and body weight were measured for cats in this study (Table S1). We collected 16 fecal samples from eight overweight/obese cats (BCS ≥ 7) and eight normal cats (BCS = 5) maintained in the same research environment (Fig. 1A and Fig. S1; see Materials and Methods). WGS metagenomic sequencing of the fecal DNA generated 1.8 billion 150 bp reads (or 271 Gbp reads). Of these, 2.21% are adapter sequences or low-quality bases, 15.21% are host sequences from the feline genome, and 0.04% are viral reads (Table S2). After removing these non-microbial reads, we performed *de novo* metagenomic assembly using 16 samples combined for a feline reference gut microbiome. The non-redundant assembly contains 355,573 microbial contigs, with a total length of 961,105,174 bp (N50 = 11,097 bp). When filtered metagenomic sequences were aligned to this feline gut microbial reference assembly for each sample, the average mapping percentage was 82.7% (Table S2) with a mean coverage depth of 282×. A total of 1.14 million non-redundant microbial genes were identified from the reference contigs. Rarefaction analysis of non-redundant genes revealed a curve approaching saturation (Fig. S2A). The number of bacterial species discovered in these metagenomes was also saturated, suggesting sufficient sequencing coverage and samples size (Fig. S2B).

### High blood glucose levels and insulin resistance were associated with feline obesity.

The eight obese cats in this study were on a similar diet to lean cats, based on major nutrients and fiber content (see Materials and Methods). Before they became obese, their body weight ranged from 3.20 to 4.10 kg (Table S1). After *ad libitum* feeding, these animals had a mean body weight of 6.20 kg at the time of fecal sample collection (Table S1), which was significantly heavier (Fig. 1B; *P < *0.001, Mann-Whitney U test). Cats in the normal body weight group were from the Scott-Ritchey Research Center breeding colony housed in the same facility, and they were significantly lighter than the obese cats at the time of fecal sample collection (Fig. 1B; *P < *0.001, Mann-Whitney U test). Blood glucose levels of these obese cats were 192.4 ± 59.3 mg/dL (ranging from 127 to 285 mg/dL), which were all above the reference interval for cat blood glucose levels determined by Auburn University College of Veterinary Medicine Clinical Pathology Laboratory (Table S3 and Fig. 1C). Serum insulin levels were also measured, and the homeostasis model assessment of insulin resistance (HOMA-IR) was 3.16 ± 0.72 (ranging from 2.44 to 3.89; Fig. 1C), which were also higher than the population-based reference interval of HOMA-IR in healthy lean cats (0.4~2.1) (6). Therefore, the eight obese cats had significantly elevated blood glucose levels with demonstratable insulin resistance at the time of fecal sample collection.

### Lack of significant sex or age effects on gut microbiome within the normal cat group.

**FIG 2:**
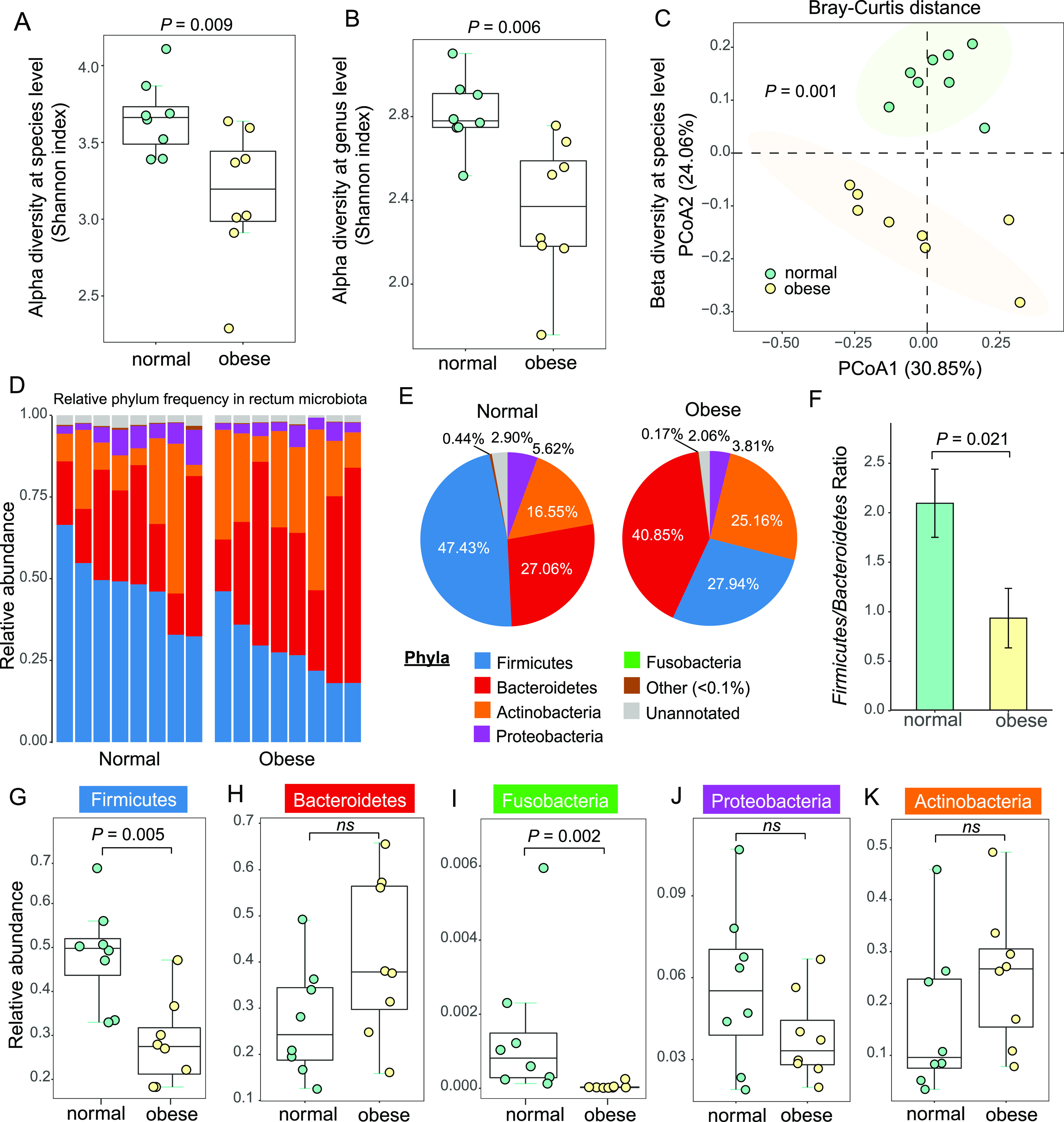
Significant changes of microbial diversity and phylum-level composition in cat gut microbiota. (A to B) Box plots of alpha diversity in normal (green) and obese (yellow) cat microbiota at the species (A) level and genus (B) level, measured using the Shannon index. (C) The PCoA plots of beta diversity between normal and obese rectum microbiota using Bray-Curtis distance. Statistical significance was assessed using permutational multivariate analysis of variance (PERMANOVA). (D) Bar plot of phylum-level relative frequency in normal and obese cat microbiota. (E) Pie charts of the phylum-level composition in normal and obese cat gut microbiota. (F) Bar plot of the Firmicutes-to-Bacteroidetes ratios in normal and obese cat gut microbiota. (G to K) Box plots of frequency for the five most abundant phyla: Firmicutes (G), Bacteroidetes (H), Fusobacteria (I), Proteobacteria (J), and Actinobacteria (K). Statistical significance was determined by the Mann-Whitney U test.

In the normal cat group in this research, four male and four female participants were included, with ages ranging from 4 months to 6 years (Table S2). To determine whether there were significant differences in the microbiome composition between sex and age groups, we performed principal coordinates analysis (PCoA) and discovered no significant effect of sex (Fig. S3A; *P = *0.473, PERMANOVA test) or age (Fig. S3B, *P = *0.468, PERMANOVA test) on the cat gut microbiome composition. Compared with the obese cat microbiota from 6-year males, the normal cats formed a cluster, which was well separated from the obese cat microbiota (Fig. 2C; *P = *0.001, PERMANOVA test). These results justified the inclusion of these eight cats in the normal body weight group.

### Significant reduction in microbial diversity in obese cat gut microbiota.

A total of 92.6% of the cat gut microbial contigs were taxonomically classified at the superkingdom level, among which bacteria account for 99.5%, with the remaining 0.5% from archaea and viruses. At lower taxonomy levels, 61.7% and 54.7% of the reference contigs were assigned to genus and species, respectively (Data set S1 and S2). Alpha diversity measured by the Shannon index showed a significant reduction in obese cat microbiome compared to normal cats, at the species level (Fig. 2A; *P = *0.009, Mann-Whitney U test) and genus level (Fig. 2B; *P = *0.006, Mann-Whitney U test). This result indicated a substantial reduction in gut microbiome complexity in obese cats compared with normal cats, suggesting dysbiosis in the obese microbiota. PCoA plot of beta diversity between normal and obese cat gut microbiomes using Bray-Curtis distance showed significant separation between these two groups at the species level (Fig. 2C; *P = *0.001, PERMANOVA test). The diversity analyses identified distinct patterns of gut microbiota in obese cats and normal cats.

### Phylum-level characterization of feline gut microbiota revealed a significantly lower Firmicutes-to-Bacteroidetes ratio in obese cats.

Among the assembled microbial contigs, 87.2% were taxonomically classified at the phylum level (Data set S3). Almost 98% of the microbes belong to the top 5 phyla, including Firmicutes, Bacteroidetes, Actinobacteria, Proteobacteria, and Fusobacteria (Fig. 2D). The most dominant phylum in normal cat gut microbiota was Firmicutes (47.4%), and the second was Bacteroidetes (27.1%), which was consistent with previously reported in 16S rDNA metagenomic studies (53) (63.3% Firmicutes and 27.6% Bacteroidetes; Fig. S4A). In obese cats, the most abundant phylum was Bacteroidetes (40.9%), followed by Firmicutes (27.9%) (Fig. 2E). This dramatic shift from Firmicutes to Bacteroidetes resulted in a significantly lower Firmicutes-to-Bacteroidetes ratio (2.10 to 0.94) in obese cat microbiota (Fig. 2F to H; *P = *0.021, Mann-Whitney U test). Another phylum, Fusobacteria, which accounted for 0.3% of the normal gut microbiome, was also depleted in obese cats (Fig. 2I; *P*-adj = 0.002, Mann-Whitney U test). No significant changes were detected at the phylum level for Proteobacteria or Actinobacteria (Fig. 2J–K; *P*-adj > 0.05, Mann-Whitney U test).

### The top 20 most abundant bacterial genera distinguish the normal and obese cat gut microbiota.

**FIG 3:**
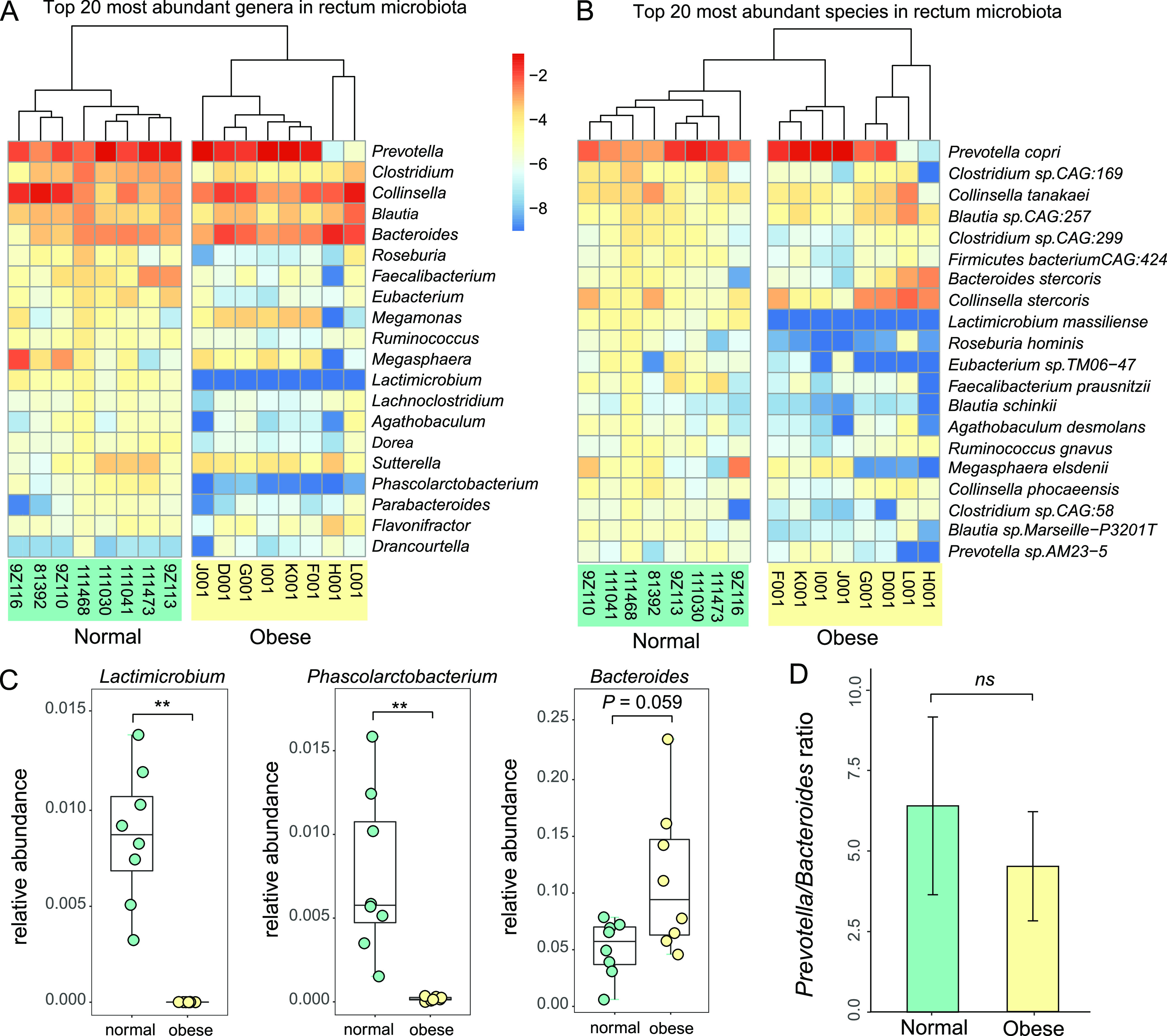
Top 20 abundant bacterial genera and species in cat gut microbiota, and their relationship to cat obesity. (A, B) Heatmap of relative frequency for the top 20 most abundant bacteria genera (A) and species (B). The taxa were rank-ordered with the most abundant taxon on the top. (C). Box plots of relative frequency for three top 20 genera that exhibit significant abundance differences between normal and obese cat gut microbiota: *Lactimicrobium*, *Phascolarctobacterium*, and *Bacteroides*. (D). Bar plot of *Prevotella*-to-*Bacteroides* ratios in normal and obese cat gut microbiota.

The top 20 genera accounted for approximately 70% of total abundance (Table S4 and Data set S1). The eight normal and eight obese cat microbiomes formed two distinct groups when unsupervised clustering was performed using the relative abundance of the top 20 genera (Fig. 3A), indicating microbial composition differences occurred at the most abundant genera level. *Prevotella* was the most abundant genus (24.3%; Fig. 3A and B and Table S4 and S5), and the relative proportions of the top 20 genera were highly correlated with the previous 16S rDNA sequencing studies (Fig. S4B; Spearman’s ρ = 0.703, *P = *0.003, Spearman’s Rank Correlation test). Of the top 5 genera, *Bacteroides* increased in the obese cat gut microbiota with a marginal statistical significance (Fig. 3C; *P*-adj *=* 0.059, Mann-Whitney U test), which may explain the overrepresentation of Bacteroidetes at the phylum level (Fig. 2E). Two Firmicutes genera were significantly altered in the obese cat gut microbiome: *Lactimicrobium* and *Phascolarctobacterium* accounted for 0.87% and 0.75% respectively in normal cats, but were not found (<0.0005%) in obese cats (*P*-adj = 0.006; Fig. 3C and Table S6). This result was consistent with the decreased abundance of Firmicutes in the obese microbiome at the phylum level (Fig. 2E). The *Prevotella*-to-*Bacteroides* ratio, which was reported to predict body weight and fat loss potential in humans (57), showed no significant change in obese and normal cat gut microbiota (Fig. 3D; *P > *0.05, Mann-Whitney U test).

### Linear discriminant analysis revealed the most featured bacterial families, genera, and species in normal versus obese cat gut microbiota.

**FIG 4:**
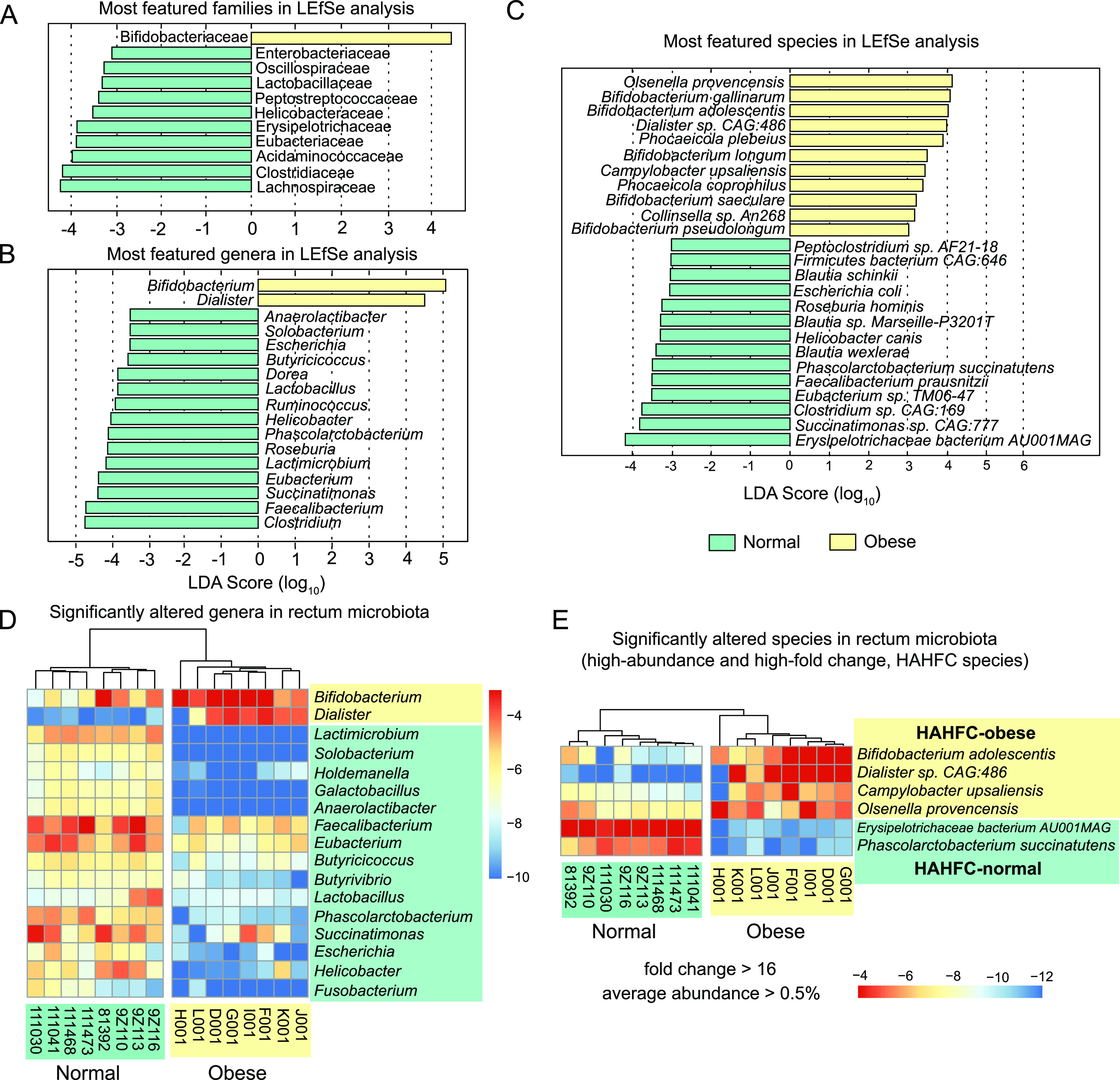
Significant differences in taxonomic abundance that discriminate normal and obese cat gut microbiome at the family, genus, and species levels. (A to C) Linear discriminant analysis (LDA) scores of top featured microbial families (A), genera (B), and species (C) in normal (green) and obese (yellow) cat gut microbiota. Taxa with an LDA score greater than 3.0 were included in these plots. (D) Heatmaps of the relative frequency for significantly (FDR < 0.10) altered genera in normal (green) and obese (yellow) cat gut microbiota. Genera with an average frequency of at least 0.1% and a minimum absolute value of log_2_ fold change (log_2_FC) of 2 were included in the plot. (E) High abundant bacterial species (relative abundance > 0.5%) with high-fold change (>16) between normal (green) and obese (yellow) cat gut microbiota. Four species enriched in obese microbiota (HAHFC-obese) and two species enriched in normal gut microbiota (HAHFC-normal) were shown in the heatmap.

To identify the featured taxa associated with obesity, we performed linear discriminant analysis (LDA) on microbial abundance profiles at the family, genus, and species levels. At the family level (Data set S4), *Bifidobacteriaceae* was the only featured family in obese cat gut microbiota (LDA > 3.0), whereas 10 families were featured in the normal microbiome, including *Lachnospiraceae*, *Clostridiaceae*, *Acidaminococcaceae*, *Eubacteriaceae*, *Erysipelotrichaceae*, *Helicobacteraceae*, *Peptostreptococcaceae*, *Lactobacillaceae*, *Oscillospiraceae*, and *Enterobacteriaceae* (Fig. 4A). At the genus level, *Bifidobacterium* and *Dialister* were the most featured obese genera to distinguish from normal cat microbiota (Fig. 4B). The normal cat microbiome featured 15 genera, 14 of which belonged to the most featured families except *Succinatimonas*, in the family of *Succinivibrionaceae* (Fig. 4A and B). The significance was driven by *Succinatimonas CAG:777*, which was the second most featured species (Fig. 4C). We identified 11 featured bacteria species in the obese microbiome (LDA score > 3; Fig. 4C), including seven Actinobacteria in the genera of *Olsenella*, *Bifidobacterium*, *Collinsella*, two Bacteroidetes (*Phocaeicola*), a Firmicutes species *Dialister* sp. *CAG486*, and a Proteobacteria Campylobacter upsaliensis. In contrast, 11 Firmicutes and three Proteobacteria species were featured in the normal gut microbiome (Fig. 4C), including the species in the top 20 genera we identified in Fig. 3A. Our results further confirmed that the normal and obese cat gut microbiota have distinct taxonomical signatures.

To determine the degree of abundance changes in the obese microbiome, we performed pairwise nonparametric tests to identify significantly altered taxa based on relative abundance (see Materials and Methods). At a false discovery rate (FDR) of 10% and relative abundance of 0.5% or higher, 17 genera have a log_2_ fold change greater than 2 (Fig. 4D). Also found in the list were 14/17 significant featured genera with LDA score > 3 (Fig. 4B), and they were the most important genera that discriminate between normal and obese cat gut microbiomes. High-abundance, high-fold change (HAHFC) marker species were filtered according to the criteria of 16-fold change and average abundance of 0.5% or higher. Six bacterial species were selected for further analysis and validation (Fig. 4E).

### MAG of the most featured species in LDA analysis - a previously uncharacterized *Erysipelotrichaceae bacterium AU001MAG*.

**FIG 5:**
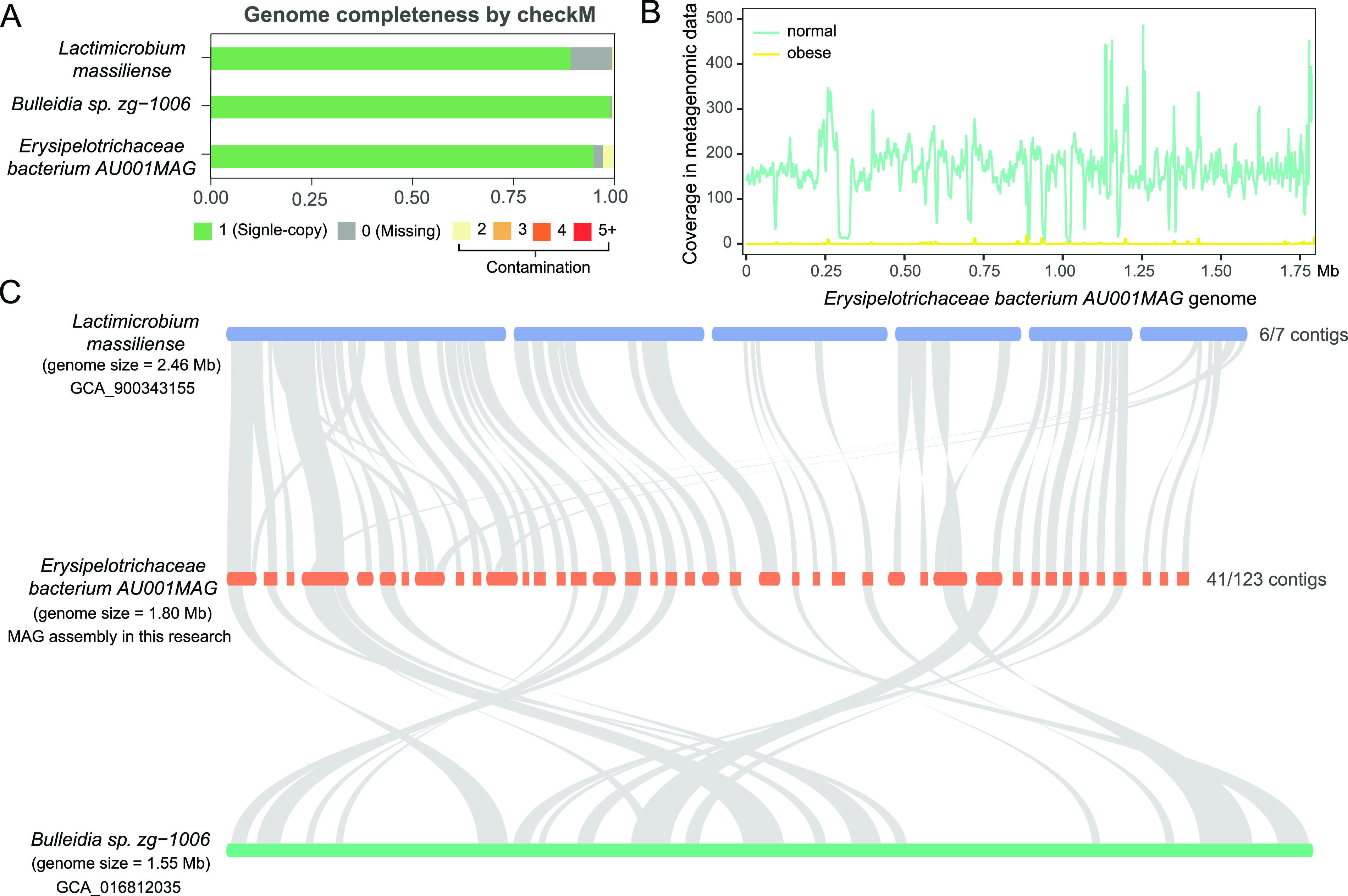
MAG genome quality assessment, normal and obese microbiota coverage, and syntenic analysis of the most featured species in obese cat gut microbiome, *Erysipelotrichaceae bacterium AU001MAG*. (A) Genome completeness of *Lactimicrobium massiliense*, *Bulleidia* sp. *Zg-1006*, and *Erysipelotrichaceae bacterium AU001MAG* assessed by checkM, showing the fraction of single-copy, missing, and contaminated genes. (B) Sliding window plot of the average coverage depth of *Erysipelotrichaceae bacterium AU001MAG* in normal (green) and obese (yellow) metagenomic data. (C) Syntenic region plot of *Erysipelotrichaceae bacterium AU001MAG* with its two most related species, *Lactimicrobium massiliense* and *Bulleidia* sp. *Zg-1006*.

**FIG 6:**
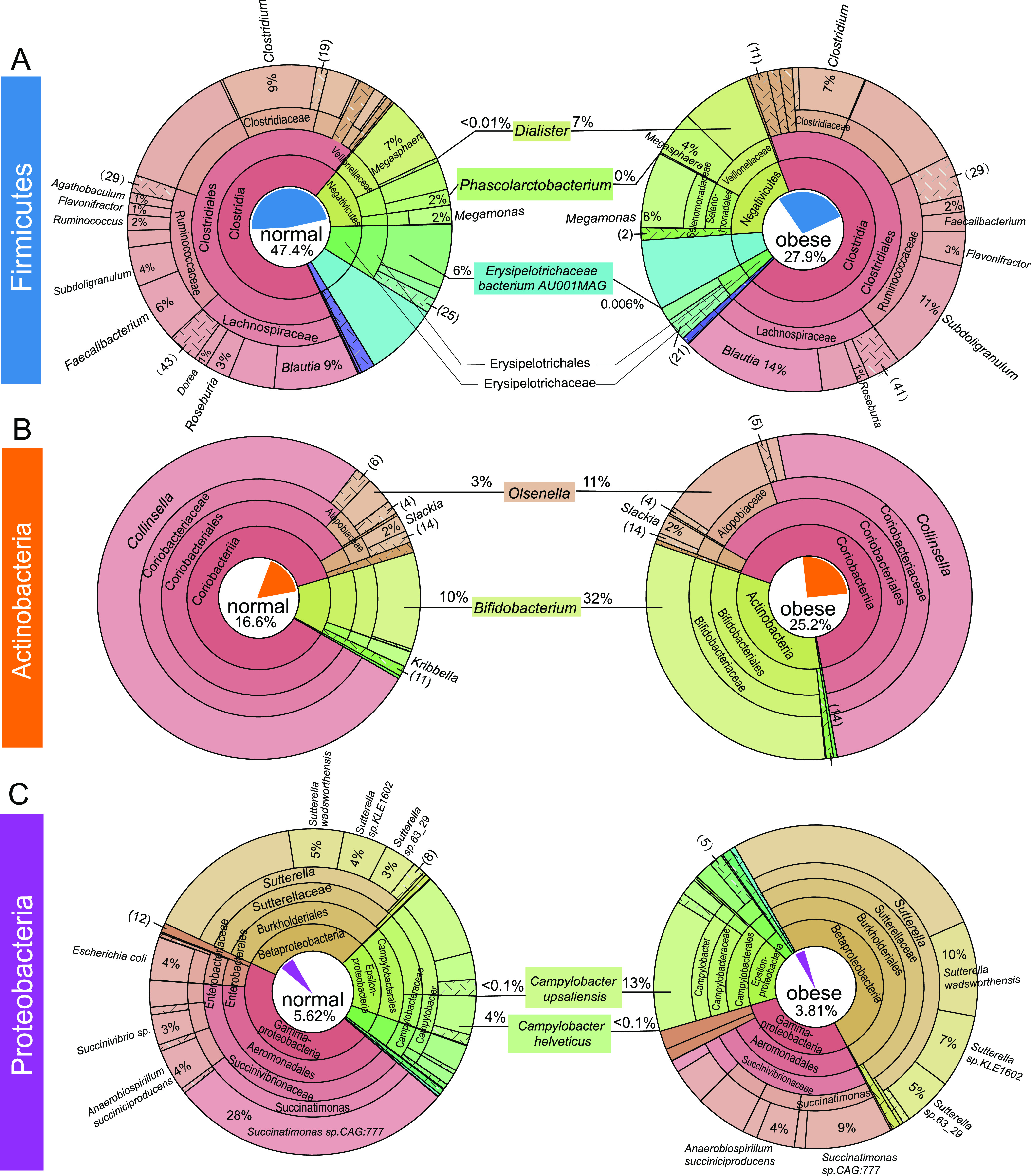
Krona plots reflecting the phylogenetic relationship and composition changes in Firmicutes, Actinobacteria, and Proteobacteria. Annotated taxonomy units within the phyla of Firmicutes (A), Actinobacteria (B), and Proteobacteria (C) were visualized in terms of relative abundance and taxonomic hierarchy for normal (left) and obese (right) cat gut microbiome. Different taxonomic terms are color-coded, and the composition percentages are labeled at the genus level (A, B) or the species level (C). The area in the chart is proportional to the relative abundance. The proportions of each phylum in the normal and obese microbiome were represented in a pie chart in the center of the circle.

The most featured species in the normal gut microbiome (LDA score > 4; Fig. 4C) was initially annotated as *Lactimicrobium massiliense* (Fig. 3B), and its reference genome sequenced strain was discovered in human breast milk from a healthy lactating mother (58). However, when the metagenomic reads were aligned to its reference assembly (GCA_900343155), the mapping rate was poor with only 82% average nucleotide identity, suggesting that this OTU in the cat gut microbiome was a different uncharacterized species in the same family of Erysipelotrichaceae (59). The metagenomic reads from this novel species were also misannotated as another closely related Erysipelotrichaceae species *Bulleidia* sp. *zg-1006* (78% sequence identity). Using the metagenomic assembly approach, we assembled a MAG genome of 1,798,709 bp in length, consistent with a single species (123 contigs with N50 = 25,047 bp and 1,657 protein-coding genes annotated). The checkM genome completeness was 96.2% (Fig. 5A), which was comparable with the two related species *Lactimicrobium massiliense* (99.1%) and *Bulleidia* sp. *zg-1006* (86.7%). We concluded that the MAG assembly of this species was nearly complete and named it *Erysipelotrichaceae bacterium AU001MAG*. This species was also the most enriched species in the normal gut microbiome (log_2_FC = 10.8, FDR = 0.01, Mann-Whitney U test, same below; Fig. 4C, 6A, and Table S7), with an average depth of >200 across the entire genome in the normal microbiome but zero coverage in the obese microbiome (Fig. 5B). *Erysipelotrichaceae bacterium AU001MAG* was the second most abundant bacterial species in the cat gut microbiome (3.1% in the normal microbiome), just trailing the most abundant species Prevotella copri (12.9%; Table S5). Gene annotation-based syntenic analysis revealed that *Erysipelotrichaceae bacterium AU001MAG* contigs could be anchored to ~2/3 of the *Lactimicrobium massiliense* genome, and most of the gene orders were conserved (Fig. 5C), suggesting that *Lactimicrobium massiliense* was the closest genome-sequenced species in the NCBI database. In contrast, *Bulleidia* sp. *zg-1006* had fewer syntenic regions and more genome rearrangement events (Fig. 5C).

### Hallmark of the obese cat gut microbiome—dramatic increases in abundance of *Bifidobacterium* sp., *Dialister* sp., *Olsenella provencensis*, and Campylobacter upsaliensis.

**FIG 7:**
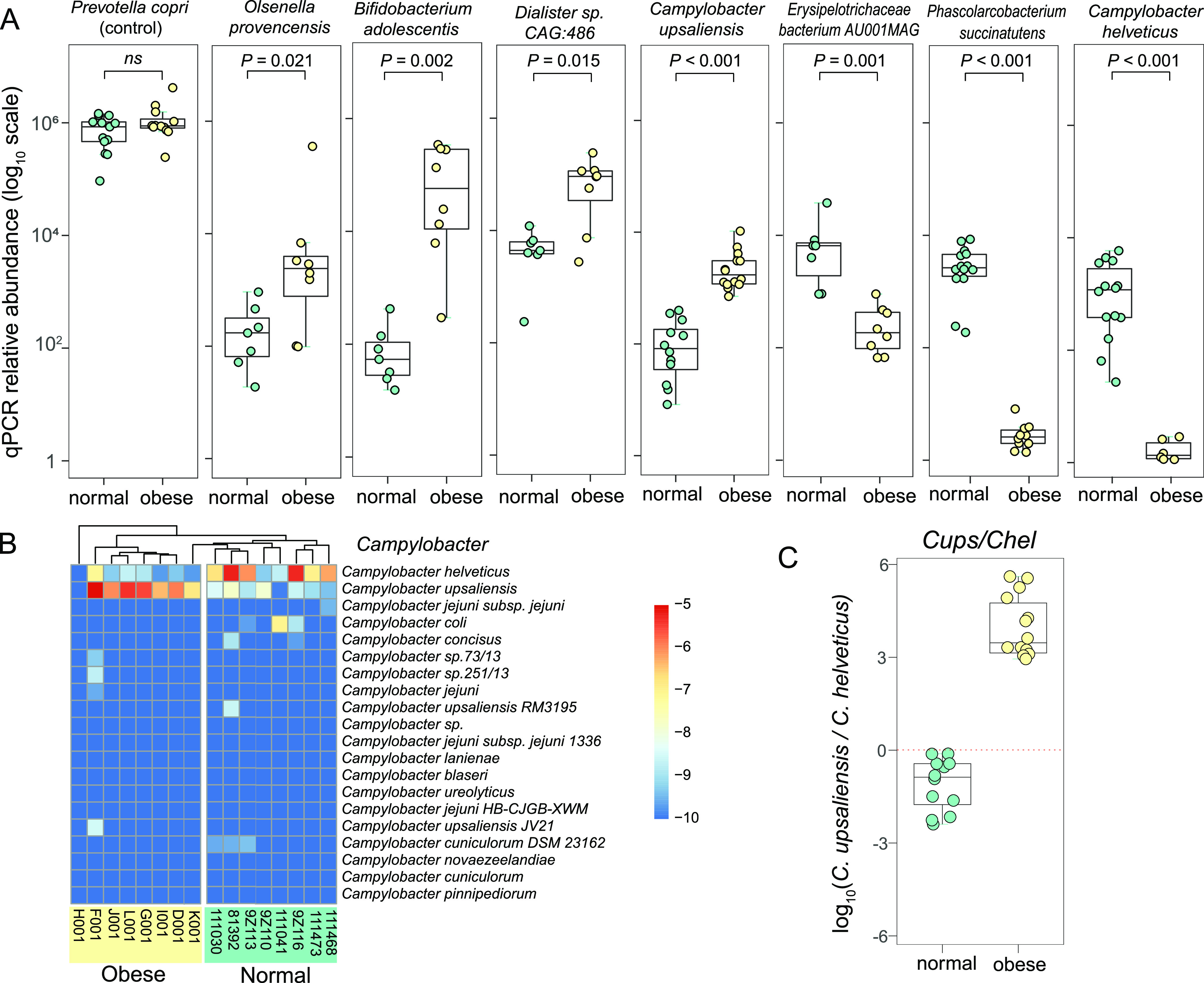
Quantitative PCR validation of seven indicator bacterial species enriched or depleted in the obese cat gut microbiome. (A) Box plots of log_10_ scale qPCR relative abundance of Prevotella copri (control), *Olsenella provencensis*, Bifidobacterium adolescentis, *Dialister sp.CAG:486*, Campylobacter upsaliensis, *Erysipelotrichaceae bacterium AU001MAG*, *Phascolarcobacterium succinatutens*, and Campylobacter helveticus in normal (green) and obese (yellow) samples. (B) Heatmap of relative frequency for the top 20 most abundant Campylobacter species. (C) Box plot of log_10_ scale relative abundance ratio of Campylobacter upsaliensis over Campylobacter helveticus in normal (green) and obese (yellow) cat gut microbiome.

A total of 400 bacteria species were significantly enriched in the obese cat gut microbiome, at an FDR of 10% and log_2_ fold change of 2 or more (Data set S5). Of these, many had an extremely low relative abundance in both groups, which were unlikely to be relevant to the disease. Eighteen obese-enriched species had a relative abundance of 0.05% or higher in the obese microbiome (Table S8), including nine Actinobacteria, four Bacteroidetes, four Firmicutes, and one Proteobacteria. Among them, four species had high abundance in the obese cat gut microbiome (>0.5%) with extremely high fold increase (fold change >16), which were defined as HAHFC-obese species (Fig. 4E and Fig. 7). As a species in one of the two most featured genera in the obese microbiome (Fig. 4B), *Dialister* sp. *CAG:483* accounted for less than 0.001% in the normal microbiome and 1.935% in the obese microbiome, with an over 1,500-fold increase (FDR = 0.04; Table S8). *Dialister* is a Firmicutes genus in the class of Negativicutes. Although we observed an overall reduction of Firmicutes in obese cat gut microbiota (Fig. 2E), the proportion of Negativicutes in Firmicutes increased from 14% to 20%, as shown in the Krona plot (Fig. 6A), which was partly driven by a dramatic increase of the *Dialister* genus from 0.003% to 7% in Firmicutes in the obese cat gut microbiome (Fig. 6A).

The number of Actinobacteria species dominated the obese cat gut microbiome enriched species (Table S8). Bifidobacterium adolescentis is the second most enriched species (FDR = 0.02, LDA score > 4; Fig. 4C and E) with a fold change of over 50, accounting for 2.11% of the obese cat gut microbiome (Table S8 and Fig. 7). *Bifidobacterium* was the other featured genus in the obese cat gut microbiome, and six species were significantly overrepresented (log_2_FC > 1.5, FDR < 0.10), including B. adolescentis, B. longum, B. pseudolongum, *B. pullorum*, *B. pullorum subsp. Gallinarum*, and *B. pullorum subsp. Saeculare* (Fig. S5 and Data set S1). Collectively, these species caused an increase of the *Bifidobacterium* genus and the Bifidobacteriaceae family from 10% to 32% in Actinobacteria (Fig. 6B), serving as a major signature of the obese cat gut microbiome (Fig. 4A, B). The other two HAHFC-obese species were *Olsenella provencensis* (Actinobacteria) and Campylobacter upsaliensis (Proteobacteria). *Olsenella provencensis* was the most featured species (Fig. 4C, E), with a 20-fold increase in the obese microbiome from 0.11% to 2.27% (Table S8 and Fig. 7). The *Olsenella* genus was also overrepresented in the obese cat gut microbiome (Fig. 6B). Campylobacter upsaliensis is a human pathogen found globally, associated with self-limiting diarrhea in companion animals and humans (60, 61). As a featured species in the obese microbiome (Fig. 4C), the abundance of C. upsaliensis is extremely low in the normal microbiome (0.020%), but a 25-fold increase was observed in the obese microbiome (Fig. 4E, 6C, and Table S8).

### Hallmark of the obese cat gut microbiome—depletion of two highly abundant species in the normal gut microbiome, *Erysipelotrichaceae bacterium AU001MAG*, and Phascolarctobacterium succinatutens.

The two top 20 genera that displayed significant differential abundance in normal versus obese microbiome (Fig. 3A and C) were driven by two HAHFC-normal species, *Erysipelotrichaceae bacterium AU001MAG* (initially annotated as *Lactimicrobium massiliense*) and Phascolarctobacterium succinatutens (Fig. 4E). They were also featured in the linear discriminant analysis at species (Fig. 4C), genus (Fig. 4B), and family levels (Erysipelotrichaceae and Acidaminococcaceae, respectively; Fig. 4A). *Erysipelotrichaceae bacterium AU001MAG* accounted for 3.1% of the normal gut microbiome, with an over 1,000-fold reduction in the obese microbiome (log_2_FC = 10.79, FDR = 0.01; Fig. 6A and Table S7). *P. succinatutens*, another highly abundant Firmicutes in the normal microbiome (0.62%), had a ~400-fold decrease in the obese microbiome (log_2_FC = 8.57, FDR = 0.01; Fig. 6A and Table S7). The depletion of these two species is a hallmark of microbiome alterations in the obese cat gut microbiome.

### Distinct metabolic pathways and CAZy families in normal and obese cat gut microbiota.

**FIG 8:**
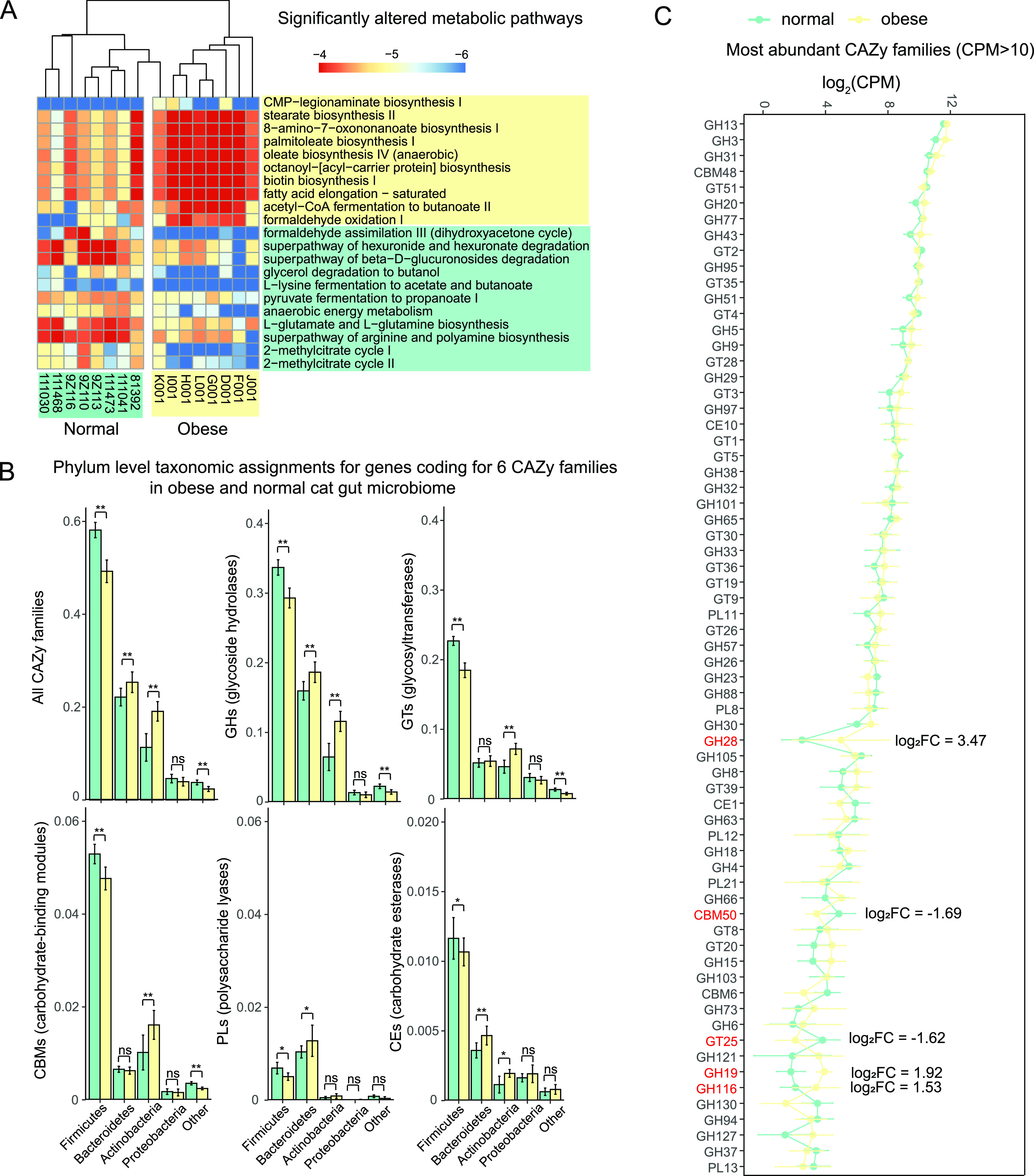
Significantly altered metabolic pathways and CAZy families in obese and normal cat gut microbiota. (A) Heatmap of the relative frequencies for significantly (FDR < 0.10) altered microbial metabolic pathways in normal (green) and obese (yellow) cat gut microbiota. Pathways with a minimum absolute value of log_2_ fold change (log_2_FC) of 1.5 were included in the plot. (B) Bar plots of percentages for phyla to which CAZyme genes from different CAZy families in normal (green) and obese (yellow) cat gut microbiota belong. (C) Line plot of CPM (mapped reads) at log_2_ scale for most abundant CAZy families (CPM > 10) in normal (green) and obese (yellow) cat gut microbiota. CAZy families with a minimum absolute value of log_2_FC of 1.5 were denoted in red.

At the microbial metabolic pathway level (Data set S7), we identified 10 pathways significantly enriched in abundance in the obese cat gut microbiome (log_2_FC > 1.5, FDR < 0.1), while 11 pathways were significantly depleted (log_2_FC < −1.5, FDR < 0.1; Data set S7). Among the obese microbiome enriched pathways, eight out of 10 were biosynthesis pathways. In sharp contrast, nine of the 11 obese microbiome depleted pathways were involved in degradation and fermentation (Fig. 8A). Overrepresented pathways in the obese microbiome included the biosynthesis of fatty acids (stearate, palmitoleate, oleate, and oxononanoate), the biosynthesis of biotin, acyl-carrier protein and nucleotide sugar CMP-legionaminate, as well as the saturated fatty acid elongation pathways. These pathways were mainly related to lipid biosynthesis. On the contrary, the normal microbiome was enriched for three degradation and two fermentation terms (Fig. 8A). Methylcitrate cycle I and II, as well as the biosynthesis of glutamine and arginine amino acids were also enriched compared with the obese cat gut microbiome (Fig. 8A).

As an important aspect of microbiome function, carbohydrate-active enzymes (CAZymes) are responsible for the synthesis and breakdown of complex carbohydrates in the cat gut microbiome. Based on the protein sequence homology to the CAZy database, we detected 105 CAZy families (Data set S8), which were assigned to 51 glycoside hydrolases (GHs), 38 glycosyltransferases (GTs), seven carbohydrate-binding modules (CBMs), six polysaccharide lyases (PLs), two carbohydrate esterases (CEs), and one auxiliary activities (AA). Overall, a larger number of CAZyme encoding genes were characterized in the normal gut microbiome compared to the obese microbiome, but the proportion of CAZymes in all annotated genes was higher in the obese microbiome. A total of 1.26% of the annotated genes in the obese cat gut microbiome were CAZyme encoding genes, which was significantly higher (*P = *0.01, Mann-Whitney U test) than the proportion of CAZymes in the normal microbiome (1.19%; Fig. S6A). When taxonomy abundances of CAZymes were measured by counts per million (CPM mapped reads), Firmicutes and Bacteroidetes were most abundant in CAZymes, accounting for 77.5% of all CAZyme abundance (Fig. 8B). In the obese cat gut microbiome, a significant increase of CAZyme abundance originated from Bacteroidetes was observed, whereas Firmicutes CAZymes were significantly decreased (*P < *0.01; Fig. 8B), which was consistent with the microbial composition changes at the phylum level (Fig. 2E). The top 6 CAZyme-encoding genera accounted for 32.2% of all CAZyme encoding genes, including *Bacteroides* (8.15%), *Clostridium* (5.83%), *Prevotella* (5.04%), *Blautia* (5.01%), *Collinsella* (4.46%), and *Bifidobacterium* (3.67%; Fig. S6B).

By comparing the relative abundance of CAZyme families between normal and obese microbiomes, we discovered that in the obese cat gut microbiome, Firmicutes were significantly less in GHs, GTs, CBMs, PLs, and CEs, whereas Bacteroidetes were significantly enriched for GHs, PLs, and CEs (Fig. 8B). Actinobacteria were also higher in GHs, GTs, CBMs, and CEs in the obese microbiome (Fig. 8B), suggesting a potential contribution of the carbohydrate metabolism primarily in the obese cat gut microbiome. Among the 63 highly abundant CAZy families (CPM > 500), five enzymes had a log_2_ fold change of 1.5 or higher. GT25 (log_2_FC = −1.62) and CBM50 (log_2_FC = −1.69) were significantly decreased in obese cat gut microbiome (FDR < 0.05). Three glycoside hydrolases, GH28 (log_2_FC = 3.47), GH19 (log_2_FC = 1.92), and GH116 (log_2_FC = 1.53), were upregulated in the obese cat gut microbiome with marginal significance.

## DISCUSSION

### Feline gut microbiota composition—similarity to canine and human microbiome and consistency between WGS and 16S rDNA data.

To our best knowledge, we report here the first metagenomic assembly of the feline gut microbiome using WGS metagenomic approaches. In normal lean cat gut microbiomes, Firmicutes, Bacteroidetes, Actinobacteria, Proteobacteria, and Fusobacteria were the top 5 most abundant phyla, which were also the top 5 phyla of the human and dog gut microbiome in the same order (62–64). Compared with a previous 16S rDNA study of cat gut microbiome in 2017 (53), the taxonomy abundance quantified in this study has a high correlation when the top 5 phyla and the top 20 most abundant genera were examined, suggesting that the composition of the feline gut microbiome was stable in different cat populations under similar but slightly different standard diet (Mars Petcare diet with 39.8% protein, 12.5% fat, 38.3% carbohydrate, and 2.3% crude fiber was used in Fischer 2017). This also serves as a proof of principle of our WGS metagenomic study.

### The first cat gut microbiome contigs assembly and microbial gene catalog provided sequence references and information of sufficient samples size for future studies.

In this study, we assembled 234 Gbp of high-quality microbial reads from a total of 16 metagenomes and generated a *de novo* assembly of the feline gut microbiome. The non-redundant contigs length was 961 Mbp in total, with 1.14 million predicted microbial genes.

Rarefaction analyses found that both the number of bacterial species and microbial genes were >90% saturated when *n *>* *5 samples were included, indicating that a sample size of *n *=* *6 is sufficient in future WGS metagenomic analysis of cat gut microbiomes. A sample size larger than *n *=* *6 would only have marginal benefit in identifying additional taxa. The result suggested that the sample size in this study (*n *=* *6 for each group) is sufficient and the reference assembly with 16 metagenomes has excellent completeness. On average, 83% of the metagenomic reads were aligned to our reference assembly, which is comparable with the human gut microbiome reference genome (65). Compared with the canine gut microbiome with 1.25 million predicted microbial genes (66), there were 9% fewer non-redundant genes in the cat gut microbiome. A total of 95.9% microbial genes in cat gut catalog had a phylum-level annotation, and genus/species level annotations were available for 68.9% and 62.7% of genes. The feline gut microbial gene catalog served as a comprehensive annotation set for functional studies of the microbiome.

### Potential confounding factors in comparing normal versus obese cat microbiomes.

In humans and mice, sex, age, and diet can significantly affect the gut microbiome compositions. The gut microbiota associations with feline obesity had been studied in the context of age, diet, neutering, and diabetic status (32, 48, 53, 54). In all four independent studies, obese status was discovered to influence the cat gut microbiome, but no significant effects of age, sex, diet, or neutering status were detected in previous feline 16S rDNA studies by multiple research groups. In a 2016 study using 16S rDNA PCR analysis of fecal samples from shelter cats, no significant associations were identified between bacterial groups and sex or neutering status (32). The cats were on various diets and of diverse age groups (16 cats between 10-week and 1-year, 41 between 1-year and 5-year, and 20 of unknown age). Another study in 2019 contrasting the microbiomes of diabetic and control cats found no effect of breed, sex, or age on the gut microbial communities (54). In a 2020 study comparing lean and overweight cats, no significant differences were discovered in fecal microbiomes due to sex or age or different diet groups (48). Another study published in 2017 also found no significant effects of sex or age on cat gut microbiome in adult cats (53). In this study, the obese group consists of eight 6-year-old male cats. For the normal body weight group, we enrolled three 6-year-old cats to match the obese group; two 4-month-old cats and three 8-month-old cats were also included to make it a balanced comparison. No significant differences due to age or sex were detected according to permutational multivariate analysis of variance, which was consistent with all previous 16S rDNA studies (32, 48, 53, 54). The normal and obese cats were on two different brands of standard adult cat food with similar nutritional compositions. There could be subtle gut microbiome changes due to the slight differences in the diet, but none of the minor variations between the diet are sufficient to explain the dramatic microbiome composition shifts observed between normal and obese groups. Consistent with this interpretation, the previous 16S rDNA studies confirmed that diets with similar nutrient ingredients did not affect the cat gut microbiota (32, 48), and cross-comparison between our normal cat group and the Fischer 2017 16S rDNA study revealed highly correlated microbial genera composition (Fig. S4B), despite the differences in diets. Therefore, it is extremely unlikely standard diets with similar nutrients will cause 100-fold changes in bacterial composition observed in our study, but it is still a potential limitation of this research and might decrease the statistical power. Future studies that evaluate the feline microbiome using metagenomics sequencing should consider diet as a potential variable when interpreting their findings.

### Signatures of obese cat gut microbiota—what did we learn at the microbial diversity level?

Given that the gut microbiota composition is directly relevant to the host’s digestion and energy metabolism, thorough identification of gut microbiome signatures is critical to define the medical condition of feline obesity in terms of microbiota dysbiosis. Significant differences in the gut microbiome have been reported in obese compared to lean cats using PCA analysis (32), but the qPCR approach cannot determine the microbial diversity. Another 16S rDNA metagenomic study of lean neutered/intact and obese cats identified a lower alpha diversity in lean neutered cats, and no significant grouping was detected when beta diversity was analyzed (53). In this study, we discovered a significant reduction in alpha diversity at both the genus and species levels in the obese microbiome, suggesting dramatically reduced microbial complexity, which often reflects a state of dysbiosis in the gut microbiome. The beta-diversity analysis also revealed a distinct separation of the normal and obese cat microbiomes. In addition to the taxonomy level, the reduced diversity was also observed at the gene level, in which the number of microbial genes predicted in the obese microbiome (598,349) was significantly fewer than the normal cat microbiome (912,251). Another study of the gut microbiome of diabetic cats discovered decreased gene mark richness in diabetes mellitus (DM) cats (FDR = 0.04) (54). The obese cats in this study also demonstrated significant insulin resistance, and the reduction in gene richness was in the same direction as the 2019 study (54).

### Shift from Firmicutes to Bacteroidetes in obese cat gut microbiota is in the opposite direction compared with human and mouse gut microbiomes.

Phylum-level abundance changes are directly relevant to obesity. Previous studies in humans and rodents found that the ratio of the two most dominant phyla, the Gram-positive Firmicutes over the Gram-negative Bacteroidetes, was elevated in obese individuals and may be a hallmark of obesity (29, 67–69). The validity of this potential marker was questioned subsequently by contradictory results (70–74), but this metric was still worth investigating. Interestingly, we observed an inverse pattern compared with what was reported in humans and rodents, with a significantly decreased Firmicutes-to-Bacteroidetes ratio in the obese cat gut microbiome. Bacteroidetes replaced Firmicutes as the most dominant phylum in obese cat gut microbiota. A similar pattern was also reported in a cat 16S rDNA study, in which lean neutered cats had a greater abundance of Firmicutes and a lower abundance of Bacteroidetes compared with obese neutered cats (53). Based on the current knowledge, this dramatic shift in the Firmicutes-to-Bacteroidetes ratio is likely to be unique in cats, and may serve as an indicator of microbiome health in obese and overweight cats.

### Signatures of obese cat gut microbiota—what did we learn at the microbial species level?

Previous 16S rDNA studies of the link between the gut microbiome and feline obesity were extremely informative at the phylum and genus levels, but failed to identify any individual bacteria species associated with obesity. Thanks to the resolution enabled by the WGS metagenomic sequencing, we identified hundreds of bacterial species with a significantly altered abundance between normal and obese gut microbiomes. Because many of these significant species may not be biologically relevant due to low abundances, we focused on high abundance (>0.5%) microbial species with high fold change (>16) between obese and normal cat gut microbiomes (HAHFC species). Among the six HAHFC species, Bifidobacterium adolescentis, *Dialister* sp. *CAG:486*, *Olsenella provencensis*, and Campylobacter upsaliensis were significantly enriched in the obese cat gut microbiome, whereas *Erysipelotrichaceae bacterium AU001MAG* and Phascolarctobacterium succinatutens were depleted in the obese cat gut microbiome. The significant changes in these species were validated using qPCR experiments. At the genus level, *Bifidobacterium* and *Dialister* were identified to be increased in obese/overweight compared to lean cats (FDR = 0.04 for *Dialister* and FDR < 0.0001 for *Bifidobacterium*) in a 16S rDNA study of obese cat gut microbiota (54). Our research has identified the driving microbial species in these two genera, which were the most featured genera in the obese cat gut microbiota discovered in this study. The family *Erysipelotrichaceae* was discovered to be significantly decreased (>5-fold) in obese women compared with healthy control individuals (75), which is the same direction as our results on the newly discovered species *Erysipelotrichaceae bacterium AU001MAG* in this family, suggesting it may play an important role in obesity. *Olsenella provencensis* and Campylobacter upsaliensis were not reported to be associated with obesity in any other species. Our findings of key bacterial community alterations at the species level will inform the development of probiotic treatment for weight loss therapy in cats.

### Obesity etiology from cat to human—shared significant bacterial genera between human and cat gut microbiota provide potential translational value.

In a Mayo Clinic study published in 2017, 26 participants (18 to 65 years) were enrolled in the Mayo Clinic Obesity Treatment Research Program, and the body weight was measured at the beginning and after 3 months of this program. At least 5% weight loss after 3 months was defined as success (76). Gut microbiome composition was compared between the success and failure groups. Two genera were identified with significant changes according to the LEfSe analysis (LDA score > 2), and the remaining ones were non-significant (76). Increased *Phascolarcobacterium* abundance was associated with success (*P = *0.008), and increased *Dialister* abundance was associated with failure of weight loss (*P = *0.030) (76). Strikingly, species in these two genera were among the six HAHFC species identified in this study: *Dialister* sp. *CAG486* was the most enriched bacterial species in the obese cat microbiome with a 1,500-fold increase; Phascolarctobacterium succinatutens was highly abundant in the normal cat gut microbiome, but almost missing in the obese cat gut microbiome with a 400-fold reduction in abundance. Based on the human gut microbiome study on weight loss outcomes (76) and our results in obese cats, high levels of *Dialister* may prevent body weight loss, and *Phascolarctobacterium* was associated with lean microbiomes by promoting body weight loss.

### *Bifidobacterium* in feline obesity—is *Bifidobacterium* a good choice for probiotic health supplement in cats?

*Bifidobacterium* is believed to be among the first members of microbes colonizing the human gastrointestinal tract since the infant stage. They were known to positively impact the host gut health (77). Therefore, *Bifidobacterium* is often used as probiotics to reduce gut problems such as diarrhea or constipation, and it was shown to have an impact on obesity. In rats, B. adolescentis supplementation can reduce visceral fat accumulation (78). In mouse models of high-fat diet-induced non-alcoholic fatty liver disease (NAFLD) (79) and colitis (80), B. adolescentis were shown to ameliorate the disease symptoms. Interestingly, B. adolescentis was identified as an HAHFC species in this study, with a 60-fold increase in the obese cat gut microbiome, which was in the opposite direction compared with previous rodent studies. In addition to B. adolescentis, we found that five other *Bifidobacterium* species/subspecies were also significantly increased in the obese cat microbiome. The effects on body weight were reported to be strain-dependent: B. adolescentis strains isolated from the feces of elderly human donors (Z25, 17_3, and 2016_7_2) decreased the body weight or weight gain in mice, while the strain isolated from the human newborn (N4_N3) increased the body weight in mice (81). In a recent weight management and microbiome study, cats on a high-protein, low-carbohydrate diet had decreased *Bifidobacterium* level (*P = *0.002) compared with animals on the control diet, suggesting a lower level of *Bifidobacterium* is beneficial to body weight loss (48). Taken together, higher levels of *Bifidobacterium* were associated with obesity in cats, which was different from the human and rodent studies. We need to be cautious when designing probiotic formulas for cat weight management.

### *Erysipelotrichaceae bacterium* and *Phascolarctobacterium*—beneficial bacteria for feline weight loss?

A previously uncharacterized genus *Erysipelotrichaceae bacterium* was largely depleted in obese cat gut microbiota (from 0.383% to <0.001%, FDR = 0.012), which was the most decreased species in the obese cat microbiome. Similarly, the abundance of *Phascolarctobacterium* dropped from 0.078% in normal cats to <0.001% in obese cats. We validated the dramatic decreases in both *Erysipelotrichaceae and Phascolarctobacterium* by qPCR. Moreover, the increased abundance of *Phascolarctobacterium* was proved to be associated with successful weight loss in the Mayo Clinic study (76). These two species deserve further consideration as potential probiotics for weight loss.

### Microbiome signatures in feline obesity—obese cat microbiome index Cups/Chel and a qPCR panel to detect obesity-associated microbiomes.

We detected 20 species in the genus of Campylobacter in the cat gut microbiome. A pathogenic species, Campylobacter jejuni, can colonize obese (*ob/ob*) mice with oral inoculation, and the *ob/ob* mice were extremely sensitive to C. jejuni infection (82). However, C. jejuni had low abundance in this study, and there was no significant difference between normal and obese cats. Notably, C. upsaliensis and *C. helveticus*, which were not linked with obesity before, were discovered to have significant abundance changes in the obese cat gut microbiome in the opposite direction. As an HAHFC-obese species, C. upsaliensis was almost absent in the normal microbiome (0.02%) but accounted for 0.5% in the obese cat gut microbiome. In contrast, *C*. *helveticus*, was extremely low in abundance in the obese microbiome (0.02%), but with a 12-fold increase in the normal microbiome. This inverse pattern in the normal versus obese microbiome was validated by qPCR, and the relative ratio of the two species had a much-improved discriminative power between obese and normal individuals. Therefore, we proposed to define the relative abundance of C. upsaliensis over *C*. *helveticus* as an obese cat microbiome index.

To investigate the microbiome features of feline obesity and define obesity-associated microbiomes, a total of eight microbial species were selected as an indicator panel for dysbiosis in the obese cat gut microbiome. *Prevotella* is the most abundant genus in cat gut microbiome, according to 16S studies (53) and this WGS metagenomic study. At the microbial species level, we found that Prevotella copri is the most abundant species in the cat gut microbiome, accounting for 12.9% of the entire microbiota in normal cats (Table S5). We also observed a potential trend of increasing abundance in the obese cat gut microbiome (19.6% abundance), but it was not statistically significant (*P = *0.44 and FDR = 0.67). Interestingly, Prevotella copri has a significantly higher abundance in fat pigs, and was shown to promote host chronic inflammation, intestinal permeability, lipogenesis, and fat accumulation through the TLR4 and mTOR signaling pathways (83). Our result showed a similar trend, but it did not reach statistical significance. Prevotella copri was selected as the control species for the indicator panel because of its high abundance. The panel also includes four highly enriched species in obese cat gut microbiota (Bifidobacterium adolescentis, *Olsenella provencensis*, *Dialister* sp. *CAG:486*, and Campylobacter upsaliensis), and three significantly depleted species in obese cat gut microbiota (*Phascolarcobacterium succinatutens*, *Erysipelotrichaceae bacterium AU001MAG*, and Campylobacter helveticus). This panel will serve as a cost-effective method to examine the microbiome correlates of feline obesity and can be applied in a much larger sample size.

### Fatty acids biosynthesis pathways are enriched in obesity-associated microbiota.

We discovered that fatty acid biosynthesis pathways were significantly overrepresented in the obese cat gut microbiome compared with normal cats, including biosynthesis and elongation of saturated fatty acids (SFAs). SFAs can add to the risk of cardiovascular disease by increasing the low-density lipoprotein (LDL) cholesterol levels in the serum. A study has found stearic acid-rich fat can raise the LDL/HDL (high-density lipoprotein) ratio (84). These SFAs generated by gut microbiota may contribute to lipid dyscrasia in obese and overweight cats (85). One limitation of our results is that they were merely correlations, and we do not know whether the shifted metabolic pathways caused the obesity phenotype, or the obese environment drove the microbiome changes. Further studies are needed to disentangle the causal relationships.

### Significant changes in carbohydrate metabolism on the obese cat gut microbiota.

The metabolic pathway analyses suggested that increased carbohydrate metabolism in the gut microbiome may be associated with feline obesity. The carbohydrate biosynthesis pathway of certain sugar, including CMP-legionaminate, was significantly overrepresented in the obese cat gut microbiome. A human study contrasting long-term healthy versus unhealthy diets discovered that increased degradation (or reduced biosynthesis) of CMP-legionaminate was associated with the healthy diet (86), which is consistent with the findings in this feline study. Compared with normal cats, the obese cat gut microbiome had a higher proportion of CAZymes. The elevated CAZymes were primarily driven by Bacteroidetes and Actinobacteria. When the individual CAZyme families were investigated, we discovered a significant decrease in the carbohydrate-binding module CBM50 and Glycosyltransferase GT25 in obese cat gut microbiota. GT25 belongs to GlycosylTransferase Family, and usually acts as lipopolysaccharide β-1,4-galactosyltransferase, β-1,3-glucosyltransferase, and β-1,2-glucosyltransferase. CBM50s, also known as LysM domains, mainly bind to the *N*-acetylglucosamine residues in bacterial peptidoglycans and in chitin. Three glycoside hydrolases, GH28, GH19, and GH116, were enriched in the obese cat gut microbiota. These changes in different CAZyme categories may define the microbiome functional differences in carbohydrate metabolism.

### Conclusions.

Through comprehensive analyses of normal and obese cat gut microbiota using WGS metagenomic sequencing, we report the first reference contig assembly of the cat gut microbiome and its first microbial gene catalog. This contig assembly and gene catalog provide both the reference for cat metagenome study and the essential feline microbiome toolkit for comparative analysis across mammalian microbiomes. Obese cat gut microbiome has distinct patterns compared with cats with normal body weight, including significant reductions in microbial diversity and gene numbers, a dramatic shift in phylum-level composition from Firmicutes-dominant to Bacteroides-dominant microbiome, and a number of abundant bacterial species with extremely high-fold changes (>0.5% in composition with >16-fold change). We identified the gut microbiome profiles associated with lean cat health, and a panel of marker species that indicate dysbiosis in obese cat microbiota, which may negatively impact feline health. The findings from this study will be critical to inform weight management strategies for obese cats, including evaluations of specific diet formulas that alter the microbiome composition, the development of prebiotics and probiotics that promote the increase of beneficial species and the depletion of obesity-associated species, as well as potential microbiome transplantation therapies. Bacteria identified in our study were also shown to affect the weight loss success in human patients, suggesting translational potential in human obesity.

## MATERIALS AND METHODS

### Animal selection and maintenance.

All procedures were approved by the Auburn University Institutional Animal Care and Use Committee (IACUC) with protocol number PRN 2019–3482. Animals were provided and/or maintained by the Scott-Ritchey Research Center, College of Veterinary Medicine, Auburn University. The obese group consisted of animals who participated in a study of the effects of obesity on feline health, which were obese, neutered male cats at 6 years of age (*n *=* *8). The normal group included eight lean and reproductively intact cats from the Scott-Ritchey breeding colony, ranging in age from 4 months to 6 years old (Fig. S1 and Table S1). The normal cats were fed with Hill’s Science Diet Adult Chicken Recipe Dry Cat Food with the following nutritional ingredients determined by the manufacturer (Hill’s): 35.0% protein, 21.4% fat, 35.2% carbohydrate (nitrogen-free extract), and 1.6% crude fiber. The obese cats were on the LabDiet laboratory feline diet 5003 with the following ingredients provided by the manufacturer (LabDiet): 30.5% protein, 24.5% fat, 38.1% carbohydrate (nitrogen-free extract), and 2.3% crude fiber. Both diets were standard adult cat food with very similar nutritional ingredients. No probiotics were provided to any of these cats. No antibiotic treatments were applied to any of these cats within 2 months prior to the study. The cats were not experiencing any stress prior to the fecal sample collection either.

### Morphometrics, blood glucose, and insulin measurements in obese cats.

Cats were sedated to effect using medetomidine, ketamine, and butorphanol administered intramuscularly. Body condition score was evaluated using the World Small Animal Veterinary Association criteria for cats (87, 88). Whole blood glucose was evaluated immediately using the AphaTrak 2 monitoring system (Zoetis, MI). Serum was separated from clotted whole blood by centrifugation at 800 g for at least 15 min and was frozen at −80 C until needed. Serum insulin was determined using a commercially available ELISA kit specific to cats (Mercodia Inc., NC) (89). The homeostatic model assessment for insulin resistance (HOMA-IR) was calculated as the basal glucose and insulin concentration product, divided by 22.5 (6).

### Fecal sample collection and microbial DNA extraction.

Fecal samples were collected under sedation immediately after blood collection to prevent interference of epinephrine-mediated hyperglycemia (Table S1). Plastic fecal loops were coated with mineral oil and inserted into the rectum and descending colon of the cats until an adequate amount of feces was collected. The samples in this study reflect the fecal composition of the rectum and descending colon, which is representative of the lower gut. We referred to the microbiota characterized in the fecal samples in this research as cat gut microbiota.

Genomic DNA samples were extracted from 200 mg fecal samples using the Qiagen Allprep PowerFecal DNA/RNA kit (Qiagen, MD) following the manufacturer’s protocols. To achieve homogeneous results, the homogenization step was performed by the Qiagen PowerLyzer24 instrument (Qiagen, MD) in the same batch. DNA and total RNA concentrations were measured by a Qubit 3 Fluorometer (Invitrogen, CA), and the A260/A280 absorption ratios were assessed using a NanoDrop One C Microvolume Spectrophotometer (Thermo Fisher Scientific, MA).

### Metagenomic sequencing, quality control, and preprocessing of metagenomic reads.

For each sample, 1.5~2 μg of DNA was fragmented by M220 Focused-ultrasonicator (Covaris, MA) to achieve a target insert size of 500 bp. WGS metagenomic sequencing libraries were constructed using NEBNext Ultra II DNA Library Prep Kit for Illumina (New England Biolabs, MA), according to the manufacturer’s protocols. Final library concentrations and size distributions were determined by LabChip GX Touch HT Nucleic Acid Analyzer (PerkinElmer, MA). The libraries were measured by qPCR before being sequenced on an Illumina NovaSeq6000 sequencing machine with 150-bp paired-end reads at the Genomics Service Laboratory at the HudsonAlpha Institute for Biotechnology (Huntsville, AL).

A total of 1.8 billion sequencing reads (or 271 Gbp reads) were obtained from 16 metagenomes (Table S2). Paired-end reads were merged to increase read length with PEAR (v0.9.11) (90). Adapter sequences and low-quality sequences were cleaned from subsequent reads using Trimmomatic (v0.36) (91). High-quality filtered reads were then mapped to the feline reference genome (GCF_000181335.3) using Burrows-Wheeler Aligner (BWA) (v0.7.17-r1188) (92) and SAMtools (v1.6) (93). The retained reads were mapped to the viral genome database downloaded from National Center for Biotechnology Information (NCBI) to remove the viral sequences (94). The remaining reads were extracted for subsequent analysis using BEDTools (v2.30.0) (95).

### Feline gut metagenome assembly and microbial gene annotation.

The filtered reads were assembled into metagenomic contigs with MEGAHIT v1.1.2 with default parameters (96). Contigs shorter than 400 bp were filtered out, and redundant contigs were removed using cd-hit-est (v4.7) (97, 98) with the criteria of global sequence identity more than 95%. Microbial genes were predicted from the assembled cat reference metagenomic contigs using MetaGeneMark (v3.38) (99).

### Taxonomy assignment and taxonomy abundance analysis.

Taxonomy assignments for these non-redundant metagenomic contigs were performed using Kaiju (v1.7.3) (100) against NCBI-NR database at superkingdom, phylum, class, order, family, genus, and species levels. 92.6% of the contigs were annotated and assigned an NCBI taxonomy ID. Among these contigs, 54.6% are annotated to the species level. The filtered PE reads from each metagenome were aligned to the assembled cat reference metagenomic contigs (Table S2). For each sample, the relative taxonomic frequencies were calculated as the number of reads mapped to the contigs in a specific taxon normalized by the total number of aligned reads (Data set S1 to S4). The top 20 most abundant bacterial genera and species were listed in Table S4 and Table S5.

### Microbial diversity analysis in normal and obese cats.

The alpha- and beta-diversity of taxonomy profiles were performed using R package vegan v2.5.7 (101). Alpha-diversity was analyzed using the Shannon index (102) at the genus level and the species level. Beta-diversity was analyzed based on the Bray-Curtis dissimilarity (103) at the species level and visualized in the format of PCoA plot using R software (104).

### Analysis of age and sex effects in the normal cat group.

Given that age and sex may potentially affect the gut microbiome composition, we performed PCoA analysis at the species level in the control group and used permutational multivariate analysis of variance (PERMANOVA) to determine significant differences between different males and females, as well as between different age groups.

### Identification of significantly altered genera or species in normal and obese cats.

To assess the statistical significance of the differential abundance of genera or species in normal cats and obese cats, Mann-Whitney U tests (105) were performed in R (Data set S5, S6). The heatmap plots were generated using R package pheatmap (v1.0.12), and the adjusted *P* values (*P*-adj) were calculated using R package qvalue (v2.22.0) (106). Genera with an average frequency of at least 0.1% and a minimum absolute value of log_2_ fold change of 2 are listed in Table S6. Species with an average frequency of at least 0.01% and a minimum absolute value of log_2_ fold change of 2 are listed in Table S7 and Table S8.

### Linear discriminant analysis in normal and obese cat gut microbiota.

Linear discriminant analysis effect size (LEfSe v1.1.1) analysis was performed via Galaxy web application (http://huttenhower.org/galaxy) with default options to determine the most featured families, genera, and species that explain the differences between normal and obese cat gut microbiota. The relative taxonomic frequencies were used as the input of LEfSe pipeline.

### Metagenomic assembly, genome completeness, and synteny analysis of a previously uncharacterized species *Erysipelotrichaceae bacterium AU001MAG*.

The genome of the uncharacterized *Erysipelotrichaceae bacterium* species was assembled from the metagenomic reads using MEGAHIT (96), and this species is named *Erysipelotrichaceae bacterium AU001MAG*. CheckM (107) was used to assess the quality of this microbial genome and the two most related species in the family of Erysipelotrichaceae, *Lactimicrobium massiliense* (NCBI assembly accession number GCA_900343155) and *Bulleidia* sp. *zg-1006* (NCBI assembly accession number GCA_016812035). The synteny analysis of these species was performed with MCscan (Python version) (108).

### qPCR validation of microbial abundance changes.

A total of eight bacterial species were selected for qPCR validation, including Prevotella copri, the most abundant species with no significant changes between normal and obese microbiota, four highly abundant species enriched in obese cat gut microbiota (Bifidobacterium adolescentis, *Olsenella provencensis*, *Dialister* sp. *CAG:486*, and Campylobacter upsaliensis), and three species enriched in normal gut microbiota (*Erysipelotrichaceae bacterium AU001MAG*, *Phascolarcobacterium succinatutens*, and Campylobacter helveticus). The qPCR primers (Table S9) were designed in Oligo 7 software (109) and synthesized by Eurofins (Eurofins Genomics Inc., KY). For each qPCR, 30 ng fecal DNA sample was mixed with PerfeCTa SYBR green FastMix, Low ROX (Quantabio, Cat No. 95072-012) in 96-well plates, and the qPCR was performed on a Bio-Rad C1000 Touch Thermal Cycler with CFX96 Real-Time PCR Detection Systems (Bio-Rad Laboratories, CA). Non-parametric Wilcoxon Rank Sum test was performed on log_10_ scale of expression values to assess the statistical significance.

### Enrichment of functional categories and pathways.

The HMP Unified Metabolic Analysis Network, HUMAnN 3.0 (110), was used to profile the abundance of microbial metabolic pathways from metagenomic sequencing data based on MetaCyc database (111). Functional annotation was performed with eggNOG-mapper (112) based on eggNOG 5.0 database (113). CAZymes were predicted using and automated carbohydrate-active enzyme annotation tool dbCAN (114). Wilcoxon rank sum tests were performed to assess the statistical significance of the differential pathways in normal cats and obese cats.

Supplemental files are available at github.com/XuWangLab/2020_feline_microbiome_sppData.

### Data availability.

The whole-genome shotgun metagenomic sequencing data is available at NCBI SRA under accession number PRJNA758898. This whole-genome shotgun metagenomic assembly has been deposited at DDBJ/ENA/GenBank under the accession GCA_022675345.1.
